# Rethinking research into metastasis

**DOI:** 10.7554/eLife.53511

**Published:** 2019-12-17

**Authors:** Peter Friedl

**Affiliations:** 1 Department of Cell Biology, Radboud Institute for Molecular Life SciencesRadboud University Medical CentreNijmegenNetherlands; 2 Department of Genitourinary MedicineUniversity of Texas MD Anderson Cancer CenterHoustonUnited States

**Keywords:** Reproducibility Project: Cancer Biology, reproducibility, replication, metascience, biochemical remodeling, tumor microenvironment, Human, Mouse

## Abstract

The partial success of an attempt to repeat findings in cancer biology highlights the need to improve study designs for preclinical research into metastasis and the targeting of cancer cells.

**Related research article** Sheen MR, Fields JL, Northan B, Lacoste J, Ang L-H, Fiering S, Reproducibility Project: Cancer Biology. 2019. Replication Study: Biomechanical remodeling of the microenvironment by stromal caveolin-1 favors tumor invasion and metastasis. *eLife* **8**:e45120. doi: 10.7554/eLife.45120

Cancer metastasis results from the escape of cancer cells from the primary tumor, followed by circulation in the blood or lymph system, and then seeding in distant organs. In 2011 researchers at the CNIC in Madrid and other institutions in Spain and the United States reported the results of in vitro experiments and experiments in mice that highlighted how the mechanical properties of the stromal cells around a tumor can influence cancer progression and metastasis (Goetz et al., 2011). In particular they reported that the expression of an intracellular protein called caveolin-1 in tumor-associated fibroblasts resulted in remodeling of the stroma in breast cancer xenografts, which led to increased metastasis.

In 2015, as part of the Reproducibility Project: Cancer Biology, Fiering et al. published a Registered Report which explained in detail how they would seek to replicate some of these experiments (Fiering et al., 2015). The results of these experiment have now been published as a Replication Study (Sheen et al., 2019). Sheen et al. confirmed that fibroblasts expressing caveolin-1 display increased extracellular matrix (ECM) remodeling in vitro, and a higher capacity for intra-tumoral stroma remodeling in vivo. Moreover, by co-implanting caveolin-1-expressing or caveolin-1-deficient fibroblasts with breast cancer cells in nude mice, they confirmed that the expression of caveolin-1 does not affect the tumor growth at the implantation site. However, in contrast to the original study, metastasis formation was not enhanced by caveolin-1 expression, though it should be noted that there were important differences between the original work and the replication. This means that we cannot draw about conclusions about the reproducibility or otherwise of the original findings about metastasis. It is worth looking at these differences to see what we can learn for future studies.

Experiments with mice and other animals are stopped at a 'humane endpoint' to prevent unnecessary suffering. The original experiments to monitor metastasis were stopped 75 days after the breast cancer cells had been implanted in the mice. However, the tumors grew much faster in the replication, which meant that the humane endpoint was reached after just 45 days. Tumor growth and metastasis are both nonlinear processes, with long periods of relatively slow growth being followed by periods of rapid growth. It is possible, therefore, that the low levels of metastasis seen in the replication are due to the duration of the experiments being significantly shorter than the original experiments.

So what lessons might we take away from this Replication Study? First, the differences between the two studies with respect to the link between ECM remodeling in the primary tumor site and metastasis might indicate that factors other than ECM alignment have an influence on the outcome. In particular, the orientation of the ECM relative to the tumor may be important: it is known that the invasion of neighboring tissue by cancer cells, metastasis and poor prognosis are all supported when the ECM is perpendicular to the tumor surface, whereas a capsule-like orientation of the ECM parallel to the tumor surface can limit tumor cell evasion (Figure 1A; Conklin et al., 2011; Park et al., 2019).

**Figure 1.**
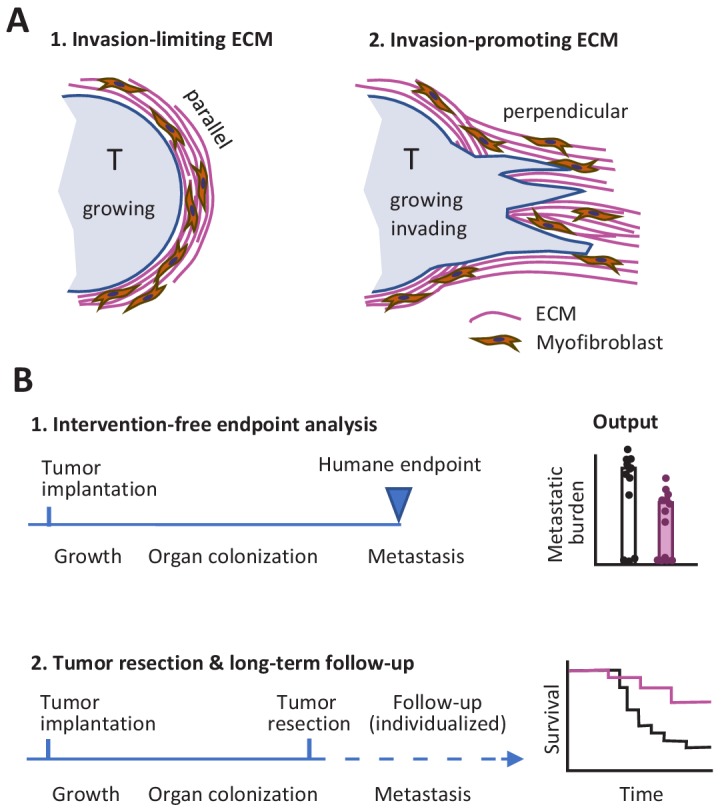
Studying tumor invasion and metastasis. (**A**) The orientation of the extracellular matrix (ECM) and fibroblasts in the vicinity of a tumor (pale blue) has an influence on the development of the tumor. A capsule-like orientation will limit the escape of cancer cells from the tumor (1), whereas a perpendicular orientation will encourage escape and the invasion of nearby tissue (2). The orientation of the ECM and the fibroblasts can be quantified by image processing. (**B**) Experiments in which cancer cells are implanted into an animal have to be stopped when the primary tumor reaches a certain size to prevent unnecessary suffering (1). This can limit the data that can be collected on other aspects of cancer, such as metastasis. An alternative approach is to monitor each animal individually and to resection a tumor when it reaches a certain size (2): this allows for longer studies, including the collection of Kaplan-Meier survival curves similar to those collected during clinical trials.

Second, it is possible to design experiments where faster-than-expected growth of the primary tumor does not limit our ability to study metastasis. This can be done by monitoring each mouse individually and resecting the primary tumor during exponential growth, before the humane endpoint is reached, while also continuing to measure metastasis and other outcomes (Figure 1B). Preclinical studies using this refined procedure have allowed researchers to measure the response of primary tumors to experimental molecular-targeted therapy and to monitor whether metastasis is affected or not for endpoints after 100 days and later (Gómez-Cuadrado et al., 2017; Miller et al., 2019). It should be noted that resection might not be required if the experimental treatment causes reliable regression of the primary tumor: the present author and co-workers recently used this approach to monitor incidence of metastasis and overall survival in tumor-bearing mice over a period of 180 days (Haeger et al., 2020).

Third, multiple experimental details that are difficult to control may affect tumor growth and metastasis. For example, tumor cells may change their growth characteristics, and grow more or less efficiently, as a result of continued culture. Other factors that influence tumor growth rates in mouse experiments include: the use of fetal calf serum to cultivate cells before implantation (van der Valk et al., 2018); the conditions under which the mice are housed (Kilkenny et al., 2010); and variation in the microbiomes of the mice (Sethi et al., 2018). It is vital, therefore, that the Methods sections of papers fully describe how the mice used in experiments were housed (including information about diet, environmental enrichment and housing temperature; see Kilkenny et al., 2010 for a full list). And in the future, molecular fingerprinting of the microbiome of animals might also be required.

In conclusion, we do not know for sure in which tumors and to what extent the expression of caveolin-1 and the subsequent remodeling of the ECM in the tumor stroma has an influence on metastasis. However, the efforts to replicate previous work in this field provide an opportunity for self-reflection, and make clear that there are strong reasons to refine workflows in preclinical metastasis research and to improve the way we carry out research into anti-cancer pathways and drug discovery.

## Note

Peter Friedl was a peer reviewer for Registered Report (Fiering et al., 2015) and the Replication Study (Sheen et al., 2019).
